# Nutrient Sensing and Redox Balance: GCN2 as a New Integrator in Aging

**DOI:** 10.1155/2019/5730532

**Published:** 2019-05-22

**Authors:** Paulina Falcón, Marcela Escandón, Álvaro Brito, Soledad Matus

**Affiliations:** ^1^Fundación Ciencia & Vida, Santiago 7780272, Chile; ^2^Biomedical Neuroscience Institute, Faculty of Medicine, University of Chile, Santiago, Chile; ^3^Center for Geroscience, Brain Health and Metabolism, Santiago, Chile

## Abstract

Aging is a complex process in which the accumulation of molecular, cellular, and organism dysfunction increases the probability of death. Several pieces of evidence have revealed a contribution of stress responses in aging and in aging-related diseases, in particular, the key role of signaling pathways associated to nutritional stress. Here, we review the possible interplay between amino acid sensing and redox balance maintenance mediated by the nutritional sensor general control nonderepressive 2 (GCN2). We discuss this new dimension of nutritional stress sensing consequences, standing out GCN2 as a central coordinator of key cellular processes that assure healthy homeostasis in the cell, raising GCN2 as a novel interesting target, that when activated, could imply pleiotropic benefits, particularly GCN2 intervention and its new unexplored therapeutic role as a player in the aging process.

## 1. Introduction

Aging is a time-dependent physiological process characterized for being dynamic and multifactorial, and contrary to the common conception, it has been proposed that aging does not start in adulthood but begins with the birth of an organism [1]. In aging, organic changes occur limiting the adaptability of the organism to the environment, leading to an increased risk of weakness, disease, and death [2]. Through the course of time, the biological functions progressively decay, accompanied by a deterioration of the ability to adapt to the metabolic stress [3].

**Figure 1:**
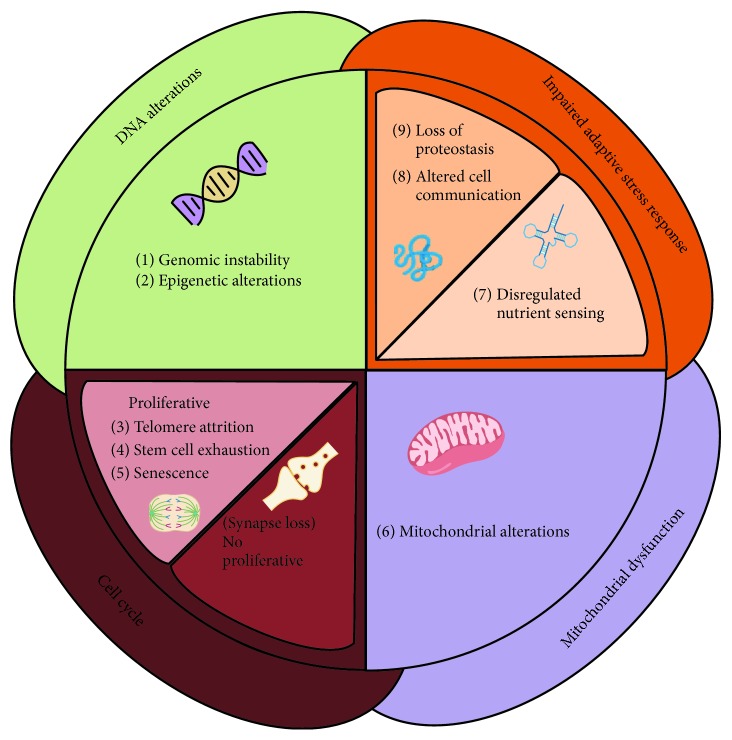
Main aging hallmarks studied in chordates. In the diagram, the nine-group aging hallmarks were grouped into four: mitochondrial dysfunction, DNA alterations (containing epigenetic alterations and genomic stability), impaired adaptive/stress response (containing loss of proteostasis and nutrient sensing deregulation), and cell cycle state dependent in function if they are differentiated or not, proliferative tissue alterations (telomere attrition, senesce), or nonproliferative cell disturbances such as synaptic loss.

The aging research field has recently born in response to the impact that it exhibits in the healthspan of a worldwide population that is getting older demographically [4]. Given the complexity of the biological phenomenon, in 2013, Lopez-Otin and colleagues [5] made the first effort in categorizing the main cellular features of aging; each one considers fulfilling three aspect criteria: (1) it manifests during normal aging, (2) its experimental aggravation should accelerate aging, and (3) its experimental amelioration should retard the normal aging process. These hallmarks, which are genomic instability, telomere attrition, epigenetic alterations, loss of proteostasis, deregulated nutrient sensing, mitochondrial dysfunction, cellular senescence, stem cell exhaustion, and altered intercellular communication [5], contribute to the aging process, and together, they establish an aging phenotype. This first attempt for shaping conceptually the aging process was fundamental in the field; however, nowadays, other authors disagree [2] regarding the applicability of the criteria of features that have only been demonstrated, so far, in proliferative peripheral tissue-associated aging such as cellular senescence and telomere attrition, in which utility in another context, namely, of a nonproliferative tissue like the brain, should be established [2]. Despite of these discrepancies, even some authors may include new mediators [6, 7]; there is a general accordance in the field about the relevance of the deregulated nutrient sensing and energy metabolism dysregulation as a key hallmark of aging [5, 8–10] (Figure 1). In general terms, the main aging hallmarks may be grouped in four sets: DNA alterations, mitochondrial dysfunction, impaired adaptive/stress response, and cell cycle-related perturbations exhibited in proliferative tissue (such as telomere attrition, stem cell exhaustion, and senesce) or nonproliferative cell disturbances such as synaptic loss (Figure 1). In this review, we will focus on stress responses evoked by nutrient scarcity and how nutrient sensing pathways could be involved in aging.

## 2. Nutrient Sensing in Aging

The importance of nutrient sensing (dys)regulation along the aging process was first demonstrated more than 80 years ago by McCay et al. [11], with the seminal observation that reduced food intake in rats, without malnutrition, extends both mean and maximal lifespan as compared to *ad libitum* fed controls. This nutritional strategy, named calorie restriction (CR), has been successfully tested in diverse eukaryotic species [12]. Thus, many efforts have been focused on delineating the molecular components linking metabolic balance induced by CR and the biology of aging, and the research has revealed a major importance of nutrient sensing in aging [13].

Nonetheless, besides the CR, lifespan extension can also be achieved by altering the diet composition; hence, nutrient and amino acid sensing mechanisms have emerged as attractive lifespan determinants. In the nutrient metabolism scene, current available evidence strongly supports the idea that amino acid (AA) sensing signaling can modify longevity [14]. The mammalian target of rapamycin (mTOR), a master growth regulator kinase, when part of mTOR complex 1 (mTORC1), can be activated by the absence of certain AAs (reviewed in [15]) and has been widely described as a key signaling pathway involved in aging [16–18]. Decreased activation of mTORC1 leads to lifespan extension in yeast, worms, flies, and mice [19], being the role of mTOR in aging extensively reviewed elsewhere [16–18]. We will focus on another, less explored in the field on aging, AA deficiency sensor, the kinase general control nonderepressive 2 (GCN2). The kinase GCN2 is a highly conserved nutrient sensor in eukaryotes at structural and functional levels [20, 21] and was identified as a critical regulator of cellular responses under AA deficiency [20, 22]. At molecular level, the mechanism of activation of GCN2 requires accumulation of uncharged tRNAs present near to the ribosome [23]. Once activated, GCN2 phosphorylates the alpha subunit of the eukaryotic initiation factor 2 alpha (eIF2*α*), causing general protein synthesis inhibition [20], an effect that promotes energy saving. At the same time, the translation of specific genes is induced, being the one coding for the activating transcription factor 4 (ATF4), the best characterized [24, 25]. ATF4 is translated using an alternative and functional open reading frame of the *ATF4* gene [26, 27] and functions to promote cell survival and adaptation during stress and AA insufficiency [25, 28]. GCN2 and its downstream activation consequences are part of the signaling pathway called the Integrated Stress Response (ISR), because it integrates cellular responses to diverse stress stimuli such as nutrient deficiency (through GCN2), viral infection (through the double-stranded RNA-dependent protein kinase (PKR)), endoplasmic reticulum stress (through the PKR-like ER kinase (PERK)), and heme deprivation (through heme-regulated eIF2*α* kinase (HRI)) [29]. The activation of the ISR in response to nutrient starvation engages adaptive changes mediated by the induction of genes necessary to produce all the amino acids [30, 31], known as the amino acid stress response (AAR). The capacity of synthesis of AA is not only a crucial step for new protein synthesis. Amino acids serve a wide variety of cellular functions. For example, amino acids supply substrates to keep the Krebs cycle activity for ATP generation and also provide reducing equivalents for maintaining redox homeostasis [25]. Thus, the consequences of AAR can be considered a strategy to cope with metabolic stress and challenges after amino acid scarcity.

The contribution of AA deficiency signaling pathways to aging, as mentioned, has been widely described based on mTOR. However, the role of GCN2 has not been extensively approached, and how GCN2 impacts the cell biology during the aging course is a question that still has no answer.

## 3. GCN2: A Nutrient Sensor That Plays a Role in Lifespan Extension

The yeast *Saccharomyces cerevisiae* (*S. cerevisiae*) has enormously contributed to the identification of mammalian genes that affect aging [32]. It was precisely in this model where there are first clues about the possible role of GCN2 in longevity [33], using the chronological aging model. In the chronological aging model, the length of time that yeast cells remain viable in a nondividing state is measured [32]. In the study by Wu et al., they showed that the extended survival induced by amino acid restriction observed in yeast was dependent on GNC2 [33]. Using another aging model in the same organism, the replicative aging, which tests the number of times a mother cell can divide and produce daughters [32], Tyler's group showed that Gcn2 (the yeast homolog of the mammalian GCN2) activation suppresses global translation efficiency, extending lifespan [34]. Considering that translation is a process that implies high energy expenditure (more than the 50% of the overall energy budget), they suggested that homeostasis is better preserved when transcript translation is reduced. Hence, reducing the translation entails a significant energy saving, which in turn could be used in restoring and maintaining cell requirements [34].

The other piece of evidence was obtained in mammals, which demonstrates that GCN2-deficient mice exhibit two main age-related effects [35] related to nutrient preference described in aging. First, GCN2 deficiency exacerbates aged mice' fat consumption at the expense of carbohydrate intake, and second, it prevented the increase in protein consumption. In this study, they suggested that GCN2 signaling might be an ancient pathway that contributes to the macronutrient selection and food preference [35]. Moreover, Kang et al. recently showed that, in response to dietary protein restriction, the lifespan of *Drosophila melanogaster* is extended, in a GCN2-ATF4 signaling axis-dependent manner [36]. The role of GCN2 as amino acid deficiency sensor has also been described in nematode *Caenorhabditis elegans* (*C. elegans*) [37], and the loss of GCN2 function is necessary for lifespan extension under nutritional stress [37]. Interestingly, for the protective role of CR on extension survival in *C. elegans*, the transcription factor PHA-4/FoxA is required [38]. PHA-4/FoxA (the nematode homolog of the mammal FoxA2) induces the expression of genes mostly involved in metabolic processes and defenses response [39]. In the context of amino AA deprivation, the extension of survival that depends on GCN2 involves the modulation of PHA-4/FoxA [37]. The other evidence is related to the protein IMPACT, which in mammals is a negative regulator of GCN2 [40]. Interestingly, the partial loss of function of IMPT-1 (yeast IMPACT Homolog 1) in *C. elegans* induces eIF2*α* phosphorylation even in a fed state. Moreover, *impt-1* knockdown exacerbates CR-induced extended lifespan and confers stress resistance [41]. This effect was dependent on other genes required for CR life extension, including *daf-16* (the yeast homolog of FOXO) and *skn-1* (the yeast homolog of Nrf 2). Thus, the discoveries found associating GCN2-dependent nutrient sensing and longevity suggest that under reduced amino acid availability, mRNA translation is inhibited and the expression of stress responses is activated, extending lifespan and improving healthspan, so far, in invertebrates. The beneficial effects of CR are also associated to the ISR activation. Still, whether GCN2 signaling or activation is impaired throughout aging in mammals is not known. Moreover, whether its functional modulation could exacerbate or attenuate the aging process is an issue that remains unclear.

Dietary methionine restriction (MR) is a proved approach to increase life span that has been shown in a variety of species (reviewed in [42]) and induce several physiological responses that confer resistance to metabolic disease [43–47]. The physiological responses to MR encompass adiposity decrease, energy expenditure increase, and thermogenic gene expression induction in the liver [48, 49]. The restriction of essential amino acids (EAAs), including methionine or leucine, limits aminoacylation of tRNAs by their cognate EAAs and induces the activation of GCN2 [50, 51]. Thus, it is possible that under MR, activation of GCN2 occurs, triggering the AAR that could be contributing to the beneficial effects observed under this specific AA diet restriction. Some of the metabolic effects of MR, including body weight reduction or elevated energy expenditure, are still present in GCN2-deficient mice [52]. GCN2 has been shown to play a pivotal role in the acute response to the essential amino acid deprivation induced by methionine restriction, while the long-term metabolic changes seem to be mediated by a GCN2-independent eIF2*α* phosphorylation [52]. These results demonstrate the complexity of the response under AA deficiency and the presence of other sensing mechanisms of the MR phenotype. However, other responses associated to GCN2 activation, described in young or old, could be driven by this amino acid deficiency during aging. For instance, GCN2 protects against hepatotoxicity after AA depletion. GCN2-deficient animals lost the capacity to engage the AAR, which is associated with hepatic triglyceride accumulation, DNA damage, oxidative stress, and inflammation [53]. In the context of ischemia reperfusion (IRI) injury, GCN2 is required for protection from renal and hepatic IRI [54]. The work from Anthony et al.'s group has revealed the protective effects of GCN2 in the central nervous system. In a mouse model of leukodystrophy, a disorder characterized by degeneration of the white matter in the brain, GCN2 is essential for protecting glial cells during amino acid deficiency [55]. Together, these results demonstrate several GCN2 protective responses that could be activated during methionine deficiency, thus mediating, in part, the beneficial effect of MR.

MR has been shown to induce drastic genetic changes, mediated by the reduction of histone methylation [56]. It is also possible to consider long-term changes induced by GCN2 activation, driven by epigenetic changes as has been proposed for ATF4 in the context of nutrient deprivation in the fetus with consequences in adulthood [28]. Regarding aging, MR has been shown to extend healthspan and lifespan in progeroid mice [57]. Even though some of the consequences of MR in mammals are quite described [58], even more, its effects on accelerated aging models is also known [57]; still, *in vivo* genetic evidence of GCN2 function in the aging process remains unclear, revealing an information gap in the nutrition sensing scenery.

## 4. Nutrient Sensing Imbalance and Its Impact in the Redox Status along Aging

Aerobic cells and organisms have developed mechanisms for dealing with the oxidative stress implicated in the cell respiration, because of the reactive oxygen species (ROS) generated as byproducts in the oxidative phosphorylation process. In homeostatic conditions, antioxidants counteract the ROS oxidative damage, which is fundamental for proper mitochondrial, thus cellular function. Throughout aging, the respiratory chain becomes ineffective, leading to electron leakage accompanied by a decrease in ATP production [59]. Thereby, the aging process results in an oxidative imbalance yielded by the increased generation of ROS and/or lessen antioxidant defenses while concomitantly cells tend to accumulate aggregated proteins and dysfunctional mitochondria [2].

Even though the relationship between mitochondrial dysfunction, oxidative stress, and aging, at first sight, might seem intuitive, the experimental evidence has not been as clarifying as expected. Remarkably, the reduction of the antioxidant defenses can accelerate the aging process, speeding up the onset of neuropathological phenotypes related to aging, such as motor dysfunction, neuronal DNA damage, and neurodegeneration in flies and mice [60, 61]. On the other hand, there exist contradictory data, particularly the findings that the increase of ROS can extend the lifespan in invertebrate models like yeast and *C. elegans* [62–64]. While the evidence in mice is intriguing as well, given that the increase mitochondrial ROS and oxidative damage do not accelerate the aging process [65, 66], the increased antioxidant defenses extend longevity [67–71].

Despite the contradictory evidence, as a consensus in the field, the proper oxidative status of the cell, constituted by the fine balance between ROS and antioxidant defenses, is critical for the healthy cell function which in turn is controlled by different regulatory processes. Interestingly, one essential signaling that lately has been shown to be able to change the cell redox status is the nutrient sensing. How nutritional sensing and oxidative stress are integrated in the cell? *In vitro* and *in vivo* studies have demonstrated that the amino acid availability impacts in the intracellular amounts of antioxidants (for instance amino acids or glutathione), resulting in oxidative stress status changes [25, 72, 73]. This new dimension of nutrition as a modulator of the cellular oxidative status has just started to be explored in mammals. Chaveroux et al. identified GCN2 as new redox regulator that prevents oxidative stress *in vivo* [72], specifically through the transcriptional control of one of the main variants of the glutathione peroxidase 1 (GPX1), thus contributing to regulate the amount of oxidized proteins (carbonyl radicals) in the liver. In the same direction, GCN2 also can impact the redox cellular status through the regulation of autophagy in an inflammatory context. Particularly, GCN2 allows the occurrence of autophagy to increase in response to inflammation, which in turn blocks the augmentation of ROS [73]. This last finding is particularly interesting on the context of the latent proinflammatory phenotype accompanying aging [74]. Thus, nutrient sensing signaling pathways triggered by GCN2 could have protective consequences in maintaining a redox balance, in the described altered immune conditions associated with aging [74].

**Figure 2:**
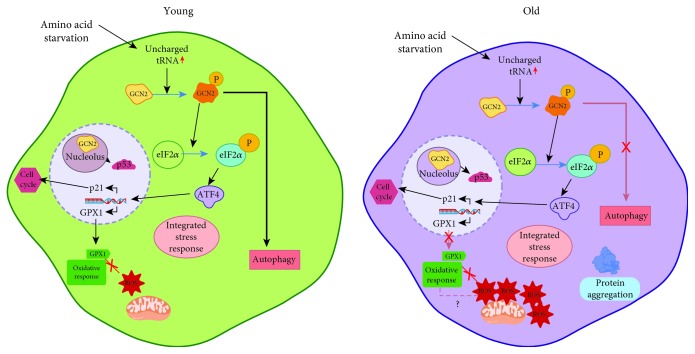
GCN2-dependent nutrient sensing along aging. Amino acid deprivation activates GCN2, a kinase that induces a nutrient stress response transcriptional program through ATF4 action. GCN2 also regulates other cell processes such as autophagy, cell cycle through p53 function, and redox status by transcriptionally modulating GPX1; this homeostatic balance can be altered throughout aging. ROS: reactive oxygen species; GPX1: glutathione peroxidase 1; GCN2: general control nonderepressive 2; eIF2*α*: eukaryotic initiation factor 2 alpha; ATF4: activating transcription factor 4.

Interestingly, GCN2 also modulates cell cycle progression through the regulation of p53 function in the nucleolus. In conditions of GCN2 deletion, p53 is activated, arresting the cell cycle and inducing canonical transcriptional targets such as p21 [75]. This recently obtained piece of evidence opens a new perspective of the role that nutritional stress controls different cell processes, particularly the integration of amino acid requirements for the cell cycle progression. Considering the above evidence, the modulation of the nutrient sensing along the aging may not only be beneficial for allowing adaptation to metabolic stress *per se* but may also contribute to balance a wide spectrum of cell processes (Figure 2). In this regard, even though the classically described function of GCN2 is to be a kinase that is activated by amino acid deprivation inducing a nutrient stress response transcriptional program through ATF4 action, GCN2 also is able to regulate other cell processes such as autophagy, cell cycle, and redox status, in which GCN2 is being a central coordinator of metabolic homeostasis that integrates the nutritional requirements in a healthy cell balance. In the aging scenery, how GCN2 integrates these cellular processes in a fine-tuned balance and the involvement of its control in the healthy aging or its contribution to the molecular mechanism of age-related disorders remains unclear (Figure 2). Along with this line, given the experimental evidence available, one could speculate regarding the benefits that could implicate the GCN2 function and manipulation throughout the aging. Considering the classic dogma that during aging the accumulation of reactive oxygen species causes cumulative cell damage and senescence, GCN2 manipulation might ameliorate the oxidation observed during aging through the transcriptional control of key enzymes involved in the oxidative counterbalance such as GXP1, diminishing the mitochondrial functional decline and bioenergetics dysregulation commonly exhibited in aging [3]. Autophagy is another process that is regulated by starvation and also by oxidative stress; albeit during aging the autophagy markers has been shown to be both up- and downregulated, autophagy has been related to both normal and pathological conditions of inflammation [76] and the defective autophagy response is one of the causes that may contribute to the accumulation of proinflammatory damage that aged tissue exhibits [74]. In this regard, considering that GCN2 has been shown to be capable of modulating inflammation through autophagy [73, 77], the GCN2 function and intervention might represent an interesting tool for attenuating the autophagy-driven proinflammatory damage observed during normal and pathological aging.

It is important to mention that angiogenesis, namely, the formation of new blood vessels by endothelial cells, even though it is considered to be an adaptive response to oxygen and/or nutrient deprivation upon ischemia or exercise, is not considered a classical aging hallmark. In this regard, different angiogenic alterations observed through the aging process have been broadly reported. Particularly, a decline in microcirculation has been described, given by a reduction in capillary density throughout aging [78]. These functional changes can be explained mostly by decreased levels of angiogenic factors in aged individuals. The abovementioned gave rise to the “angiogenesis hypothesis of aging,” in which proangiogenic therapies are proposed for ameliorating age-related symptoms (reviewed in [79]).

The canonical angiogenesis process is orchestrated by the vascular endothelial growth factor (VEGF), triggered by the hypoxia/nutrient deprivation during ischemia. Although both phenomena are difficult to dissect, hypoxia *per se* is a well-described VEGF inductor (through HIF1*α* transcriptional factor activation). Interestingly, recent studies have shown that nutrient deprivation, specifically amino acid starvation, is able to regulate angiogenesis by GCN2/ATF4 activation, in a hypoxia-independent manner, both *in vitro* and *in vivo* [80]. Particularly, GCN2/ATF4 activation shows to modulate both VEGF and hydrogen sulfide, a proangiogenic effector capable to induce the glucose intake and ATP production during endothelial cell migration [80]. Considering this piece of evidence, GCN2 stands out as a metabolic integrator able to modulate the response to stress in endothelial cells, also highlighting its therapeutic potential in ameliorating aging-related angiogenesis deficiencies, mainly improving the microcirculation condition in aged people.

## 5. Translational Potential and Therapies, Looking towards the Future

In the aging research field, the efforts now are directed to delay the aging process to diminish the vulnerability to the occurrence of age-related disorders. In terms of interventions, the dietary restriction stands out as an interesting tool. Dietary restriction, namely, the reduction of food intake without malnutrition, has the main advantage that is the less-invasive approach to be used in aged people. On the other hand, the main disadvantage of the dietary restriction is the low adherence to the treatment that might impact its therapeutic efficacy. Among these types of intervention, the most documented is the CR, which has bridged the gap between preclinical studies and human aging studies, demonstrating robustly that is able to increase lifespan in different mammalian models, including nonhuman primates (reviewed in [81]).

Particularly, amino acid deprivation has not been proved, so far, as a therapeutic strategy to modulate aging in humans. However, there exist preclinical and clinical data that show that dietary protein restriction is able to reduce the triglyceride levels in humans [82]. Considering that GCN2 has been shown to be involved in a wide variety of cellular phenomena, its manipulation is a promising tool that could implicate pleiotropic benefits. To date, the only type of intervention that has been done in order to regulate GCN2 function has been its pharmacological modulation through halofuginone (Hfg), an alkaloid originally isolated from the plant *Dichroa febrifuga* [83]. Hfg is an agonist able to activate GCN2; nevertheless, the preclinical studies using this drug have been directed mostly to the cancer research field (reviewed in [83]), evaluating how nutritional sensing modification can impact in cancer cell biology, and finally its therapeutic potential as an anticancer treatment.

However, so far, no treatment has been proven to modulate GCN2-mediated nutritional stress in aging, neither pharmacologically nor genetically.

## 6. Conclusion

The world is rapidly getting old [84], and as a consequence, aging has become an intense field of study. There is an enormous amount of studies aimed at understanding the molecular and cellular bases of aging seeking to preserve health in old stages of life. Nutritional strategies, including CR or MR, that extend life have given many clues about the signaling pathways involved in the aging process, in particular, the ones associated with nutrient sensing. Several pieces of evidence have shown that the AA deficiency sensor GCN2 and the signaling pathway triggered by its activation could be involved in the beneficial effects of restricted diets.

The GCN2 kinase has been studied in a variety of contexts including liver metabolism, innate immunity, cancer, and memory formation, among others [85]. Studies in nematode *C. elegans* have shown direct involvement of CGN2 in extending life span. However, its contribution to aging process in mammals remains unclear.

More than an AA-deficit sensing kinase, GCN2 appears to be a metabolic reprogramming controller that integrates and regulates key processes including autophagy, inflammation, and redox balance. Those are precisely contributors to aging process, and moreover, the consequences of GCN2 activation could be impacting cognitive function through neuronal and nonneuronal cells.
